# Autoimmunity as a Predisposition for Infectious Diseases

**DOI:** 10.1371/journal.ppat.1001077

**Published:** 2010-11-04

**Authors:** Mohan S. Maddur, Janakiraman Vani, Sébastien Lacroix-Desmazes, Srinivas Kaveri, Jagadeesh Bayry

**Affiliations:** Institut National de la Santé et de la Recherche Médicale (INSERM) Unité 872; Centre de Recherche des Cordeliers, Equipe 16– Immunopathology and Therapeutic Immunointervention, Université Pierre et Marie Curie – Paris 6, UMR S 872; Université Paris Descartes, UMR S 872, Paris, France; The Fox Chase Cancer Center, United States of America

Autoimmunity refers to an inappropriate immune response against self-components of the host that results in pathological conditions. Autoimmune diseases are characterized by an activation of autoreactive T and B cells, are associated in some cases with the production of pathogenic autoantibodies against self-molecules, culminating in inflammation and tissue damage. The reasons for the breakdown of tolerance mechanisms leading to autoimmunity are not clearly known. However, a combination of genetic, immunological, and environmental factors plays a critical role in the pathogenesis of autoimmunity [1]–[5].

## Autoimmunity Can Predispose to Infectious Diseases

**Figure 1:**
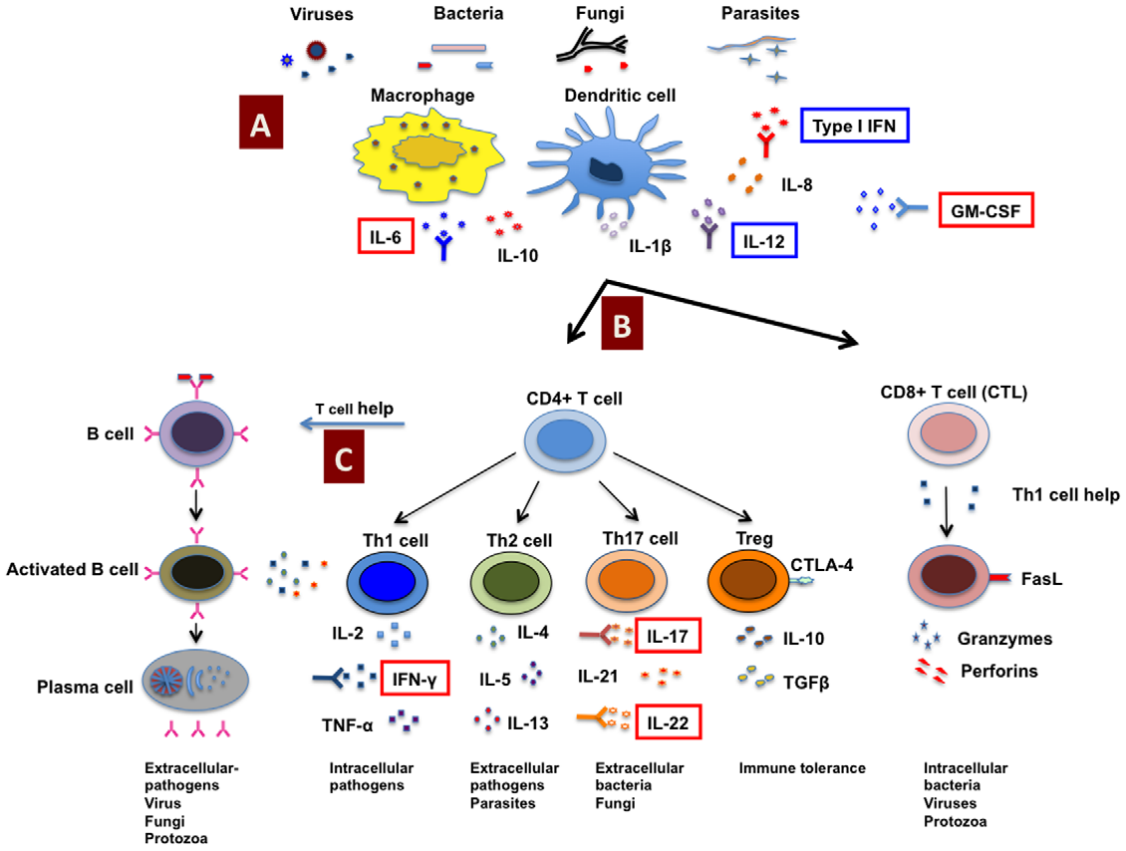
Host immune response to pathogens and predisposition to infections due to autoimmunity. Antigens from invading pathogens are recognized and presented by innate immune cells (A) such as macrophages and dendritic cells to CD4+ and CD8+ T cells (CTL) (B). CD8+ T cells recognize endogenous antigens presented by MHC class I molecules and exert cytotoxic functions upon activation. CD4+ T cells recognize antigens presented in the context of MHC class II molecules, and under the influence of innate cells and cytokine milieu, CD4+ T cells can be polarized into different subsets such as Th1, Th2, Th17, and regulatory T cells (Tregs) that secrete distinct cytokines. CD4+ T cells provide help to B cells to produce antigen-specific antibodies (C). However, due to autoimmunity, neutralizing autoantibodies can be produced against any of these key components of the immune system critical for mounting anti-microbial responses and might either predispose to an increased risk of bacterial, viral, and opportunistic fungal infections or exacerbate the ongoing infectious diseases. Indeed, in patients with infections, the occurrence of neutralizing autoantibodies against several key cytokines such as IFN-γ, IL-6, GM-CSF, IL-17, and IL-22 (highlighted in red boxes) that interfere with the host immune response to pathogens have been demonstrated. In addition, autoantibodies are also reported against type I IFNs and IL-12 that might play role in predisposition to infections (highlighted in blue boxes). CTLA-4, cytotoxic T lymphocyte antigen-4; CTL, cytotoxic T lymphocyte; FasL, Fas ligand; GM-CSF, granulocyte/macrophage–colony stimulating factor.

During the course of autoimmunity, autoantibodies that can neutralize key components of the immune system that are essential in mounting anti-microbial responses may be produced (Figure 1). These autoantibodies might either exacerbate ongoing infectious diseases or predispose the individual to an increased risk of bacterial, viral, and opportunistic fungal infections. For example, cytokines play a critical role in the process of mounting anti-microbial responses due to their ability to regulate the innate and adaptive immune systems, in polarizing T cell responses, and by acting as effector molecules. Thus, IL-12 mediates Th1 cell differentiation and IL-4 influences Th2 differentiation. IL-6, IL-21, TGF-β, IL-1β, and IL-23 are critical for the differentiation and expansion of Th17 cells. Th1 cells produce cytokines IFN-γ and IL-2, and confer protection against intracellular pathogens (viruses and intracellular bacteria such as *Mycobacterium* and *Salmonella*). Th2 cells produce IL-4, IL-5, and IL-13, and are important to clear extracellular pathogens and parasites. Th-17 cells secrete IL-17, IL-21, and IL-22, and provide protection against several extracellular pathogens, including fungi such as *Candida* (Figure 1) [6]–[9]. In addition, type I IFNs have a critical role in anti-viral immunity and in modulating T and B cell responses. Therefore, it can be conceived that the development of neutralizing antibodies against any of these cytokines as a consequence of autoimmunity affects the cellular functions and clearance of pathogens and predisposes the host to infectious diseases. This is further supported by reports of a high prevalence of infections in autoimmune patients treated with neutralizing monoclonal antibodies to inflammatory cytokines. Patients with rheumatoid arthritis, Crohn's disease, or psoriasis treated on a chronic basis with monoclonal antibodies to TNF-α are predisposed to mycobacterial, listerial, and viral infections [10]–[12].

## Specific Examples of Autoimmunity Favoring Infectious Diseases

Several reports have now demonstrated the occurrence of neutralizing autoantibodies against cytokines in patients with infections. These reports thus provide a pointer towards a previously unknown link between autoimmune responses and predisposition to infectious diseases.

A correlation between neutralizing autoantibodies to IFN-γ and mycobacterial infections has been reported [13]–[20]. Moreover, the clinical features of patients with anti-IFN-γ immunoglobulin G (IgG) are analogous to those with genetic defects in the IFN-γ/IL-12 pathway, which is characterized by progressive or disseminated infection with mycobacteria of low virulence, indicating that anti-IFN-γ IgG induces an acquired immunodeficiency state and predisposes to mycobacterial infections [13]–[20]. These anti-IFN-γ IgG neutralized IFN-γ in whole blood culture, inhibited IFN-γ-dependent phosphorylation of STAT-1 and production of TNF-α and IL-12 by normal peripheral blood mononuclear cells (PBMCs) and macrophages, and inhibited HLA-DR expression in normal monocytes [14]–[17]. In another study, one patient's serum was shown to inhibit IFN-γ-mediated upregulation of MHC class I on Jurkat cells [20]. Given the critical role of the type I cytokine pathway in the immune response to mycobacterial infections [21], these reports provide direct evidence for how anti-IFN-γ autoantibodies can affect protective anti-mycobacterial immunity.

Recurrent staphylococcal cellulitis and subcutaneous abscesses were reported in a child with autoantibodies against IL-6 [22]. These anti-IL-6 autoantibodies inhibited IL-6-mediated STAT3 phosphorylation and C-reactive protein (CRP) production in Hep3B cells. Since IL-6 is pivotal for CRP induction, these results indicated that anti-IL-6 autoantibodies contributed to the lack of CRP response in this patient during staphylococcal infections. In addition, IL-6- deficient mice have been shown to be susceptible to various pyogenic infections, including *Streptococcus pneumoniae*, *Pseudomonas aeruginosa*, and *Klebsiella pneumoniae* [23]–[26]. Interestingly, autoantibodies to IL-6 were not identified in other patients with severe staphylococcal diseases and hence suggest that anti-IL-6 autoantibodies were not generated due to molecular mimicry with *Staphylococcus aureus*. In addition, the course of clinical events in the patient was suggestive of an occurrence of anti-IL-6 autoantibodies that preceded staphylococcal infection.

Patients suffering from pulmonary alveolar proteinosis (PAP) present with neutralizing antibodies against granulocyte/macrophage colony–stimulating factor (GM-CSF) and show high mortality due to infection [27]. GM-CSF has a key role in enhancing the antimicrobial activities of neutrophils and macrophages by augmenting the expression of CD11b, an adhesion molecule that mediates neutrophil adhesion to endothelial cells, and hence promoting the recruitment of neutrophils to the site of infection; promoting the differentiation of macrophages and dendritic cells (DCs); and by priming the phagocytosis and bactericidal activities of these cells. Low levels of GM-CSF autoantibodies are present in healthy individuals. These autoantibodies are implicated in scavenging and neutralizing free GM-CSF and to regulate myeloid cell functions and GM-CSF-mediated inflammation and autoimmunity [28]. However, active PAP patients have high amounts of GM-CSF autoantibodies that impair the antimicrobial functions of neutrophils, macrophages, and the expression of CD11b [29]. In addition, these autoantibodies exist abundantly in the lungs, and by effectively blocking GM-CSF binding to its receptor, they specifically inhibit alveolar macrophage differentiation, phagocytosis, and surfactant catabolism [30], [31]. Patient-derived GM-CSF autoantibodies reproduced PAP in experimental non-human primate and murine models [29], [32], while individuals with mutations in GM-CSF receptor are also affected with PAP [33]. These results thus confirm the causal relationship between defective GM-CSF function, autoantibodies, and PAP.

Th17 cytokines are implicated in protection against fungal infections, including *Candida* at mucosal surfaces, and hence neutralizing antibodies to Th17 cytokines can predispose to fungal infections [8], [34], [35]. Interestingly, neutralizing autoantibodies against Th17 cell cytokines IL-17A, IL-17F, and IL-22 have been reported in chronic mucocutaneous candidiasis (CMC) patients with autoimmune polyendocrinopathy syndrome-1 (APS-1) or thymoma [36], [37]. Of particular importance, the autoantibody titers were high before the onset of CMC. Further, individuals with mutations in *STAT3* and *IL-12RB1* showed impaired development of Th17 cells and higher susceptibility to candidiasis [38].

In addition to the above examples, autoantibodies to type I IFNs (such as IFN-α2, IFN-ω), IL-12, and TNF-α were also identified in patients with autoimmune and rheumatic diseases and in those with chronic infections [39]. Intractable (even fatal) infections in myasthenia gravis patients with thymoma might be related to high titers of anti-IL-12 and anti-IFNα autoantibodies that can reduce an IFN-γ response with a bias towards an IL-4 response [40].

Taken together, anti-cytokine autoantibodies induce an acquired immune-compromised state that predisposes the host to infections. Although autoantibodies to several cytokines are relatively widespread, they rarely neutralize to a significant extent [39]. Further, anti-cytokine autoantibodies do not seem to have co-distribution, and cytokines do have redundant functions; hence, severe infections are not common unless as described above, and neutralizing autoantibodies are developed against specific cytokines that are key in an anti-microbial response.

In view of these findings, we suggest that patients with uncontrolled or repeated infections despite antimicrobial therapy should be considered for screening and evaluating autoimmunity. Although reported examples are of autoantibodies to cytokines, the occurrence of autoantibodies that target either molecules implicated in the recognition of pathogens (such as Toll-like receptors and lectin receptors) or antigen presenting and co-stimulatory molecules cannot be ruled out. Indeed, genetic defects or polymorphisms in pattern recognition receptors and their signaling pathways and susceptibility to infections have been reported [41]–[44].

## Enigma of Induction of Anti-Cytokine Autoantibodies

Despite the reports of anti-cytokine antibodies in several malignant or infectious diseases and their low titers in healthy individuals, the high titers are predominant in autoimmune diseases [39]. Consensually, anti-cytokine antibodies against type I IFNs, IL-12, IL-17, and IL-22 are found in APS-1 or myasthenia gravis patients associated with or without thymoma [36], [37], [45]. Here, AIRE (autoimmune regulator), a novel gene that regulates peripheral self-antigen expression in medullary thymic epithelia and DCs, is mutated, leading to disturbed self-tolerance mechanisms. Thus, APS-1 patients may display autoantibodies against type I IFNs and IL-17 cytokines as a result of impaired AIRE-dependent tolerance induction. Further, extensive work by the Meager and Willcox group provided clues toward autoimmunizing mechanisms and innate cells (plasmacytoid and myeloid DCs) in the induction of anti-cytokine antibodies to type I IFNs and IL-12 [39], [40], [45].

Therefore, it is probable that autoantibodies are produced as a consequence of infections and these autoantibodies subsequently exacerbate the infectious diseases or, alternatively, a cryptic autoimmunity develops due to unknown reasons that predispose the individual to infections. Infectious agents and vaccines are often thought to be one of the environmental factors that induce autoimmunity either by molecular mimicry, epitope spreading, bystander activation of immune system, or polyclonal activation of immune cells [2], [3]. It is thus likely that in case of chronic persistent diseases such as tuberculosis, a pathogen might trigger the autoimmune process by one of these mechanisms. Indeed, the majority of patients with autoantibodies and mycobacterial infections originated from disease-endemic areas [13]–[20]. Therefore, dissection of underlying causes of autoimmunity such as genetic polymorphisms, gene deficiency, or environmental factors might shed light on these unanswered questions.

## Therapeutic Options for Autoimmunity-Associated Infectious Diseases

Therapeutic strategies for autoimmunity-associated infectious diseases should be aimed at controlling the infection as well as inhibiting the autoimmune response: blocking autoantibody-producing B cells and neutralizing autoantibodies. In this context, a combination of anti-microbial agents and immunosuppressive treatments represents a classical line of therapy for autoimmunity-associated infectious diseases. Plasmapheresis that removes autoantibodies or supplementing exogenous cytokines (against which autoantibodies have developed) are other potential therapeutic strategies. However, such therapeutic strategies do not eliminate the source of autoantibodies, i.e., autoantibody-producing B cells and plasma cells.

Autoantibody-producing B cells can be eliminated by B cell–targeted therapies (such as monoclonal antibodies to CD20, CD19, and CD22 or to B cell-activating factor (BAFF) [46]–[48]). However, repeated cycles of B cell–targeted therapies can lead to a reduction in total immunoglobulin level and predisposition to serious infections [49], [50]. Also, these therapeutic agents do not target antibody-producing plasma cells.

In view of proven safety and efficacy in diverse autoimmune diseases, polyclonal intravenous immunoglobulin (IVIg) in combination with anti-microbial agents represents an attractive therapy for autoimmunity-associated infections [51]. IVIg targets both cellular and soluble mediators of autoimmunity and inhibits the disease by multi-pronged mutually nonexclusive mechanisms such as neutralization of anti-cytokine autoantibodies by broad-spectrum anti-idiotypic antibodies, induction of B cell tolerance, inhibition of cellular proliferation, regulation of immunoglobulin repertoire, suppression of innate antigen presenting cells and inhibition of T cell help to B cells, and expansion of CD4+CD25+ regulatory T cells, the cells that are critical for maintaining immune tolerance and to suppress autoimmunity [51], [52]. Since IVIg is obtained from pooled plasma of several thousand healthy blood donors, based on the exposure of donors to infectious diseases and vaccinations, IVIg contains antibodies to a wide range of infectious agents, and hence these anti-microbial antibodies within IVIg preparations can directly neutralize pathogens [53]. However, determining an effective dose regimen and duration of IVIg therapy needs further investigation.

In our opinion, considering all therapeutic options, a “triple” combination of anti-microbial agents, B cell–targeted therapies, and IVIg represents the most appropriate and ideal method for treating autoimmunity-associated infectious diseases. Indeed, the combination of B cell–targeted therapies and IVIg has been successfully used in several autoimmune and inflammatory diseases [54], [55].

## Accession Numbers/ID Numbers for Genes and Proteins: UniProtKB

The UniProt (http://www.uniprot.org/) accession numbers for genes and proteins discussed in this paper are IL-17A, Q16552; IL-17F, Q96PD4; IL-21, Q9HBE4; IL-22, Q9GZX6; IL-23, Q9NPF7; IL-12RB2, Q99665; IFN-γ, P01579; GM-CSF, P01587; IL-6, P05231; IL-4, P05112; IL-5, P05113; IL-13, P35225; BAFF (BLyS), Q9Y275; IFN-α2, P01563; IFN-ω, P05000; TNF-α, P01375; IL-1β, P01584; IL-12 p40 (IL-12B), P29460; IL-12 p35 (IL-12A), P29459; AIRE, O43918; STAT3, P40763; HLA-DR, O19685; CD20, P11836; CD11b, P11215; CD19, P15391; CD22, P20273.
